# Enhancing the Therapeutic Potential of Mesenchymal Stromal Cell-Based Therapies with an Anti-Fibrotic Agent for the Treatment of Chronic Kidney Disease

**DOI:** 10.3390/ijms23116035

**Published:** 2022-05-27

**Authors:** Yifang Li, Sharon D. Ricardo, Chrishan S. Samuel

**Affiliations:** 1 Cardiovascular Disease Program, Department of Pharmacology, Monash Biomedicine Discovery Institute, Monash University, Melbourne, VIC 3800, Australia; yifang.li@monash.edu; 2 Development and Stem Cells Program, Department of Pharmacology, Monash Biomedicine Discovery Institute, Monash University, Melbourne, VIC 3800, Australia; 3 Department of Biochemistry and Molecular Biology, The University of Melbourne, Melbourne, VIC 3010, Australia

**Keywords:** chronic kidney disease, fibrosis, bone-marrow-derived mesenchymal stromal cells, relaxin, wound repair, angiogenesis

## Abstract

Chronic kidney disease (CKD) affects 1 in 10 members of the general population, placing these patients at an increasingly high risk of kidney failure. Despite the significant burden of CKD on various healthcare systems, there are no effective cures that reverse or even halt its progression. In recent years, human bone-marrow-derived mesenchymal stromal cells (BM-MSCs) have been recognised as a novel therapy for CKDs, owing to their well-established immunomodulatory and tissue-reparative properties in preclinical settings, and their promising safety profile that has been demonstrated in patients with CKDs from several clinical trials. However, renal fibrosis (scarring), a hallmark of CKD, has been shown to impair the viability and functionality of BM-MSCs post-transplantation. This has suggested that BM-MSCs might require a pre-treatment or adjunct therapy that can enhance the viability and therapeutic efficacy of these stromal cells in chronic disease settings. To address this, recent studies that have combined BM-MSCs with the anti-fibrotic drug serelaxin (RLX), have demonstrated the enhanced therapeutic potential of this combination therapy in normotensive and hypertensive preclinical models of CKD. In this review, a critical appraisal of the preclinical data available on the anti-fibrotic and renoprotective actions of BM-MSCs or RLX alone and when combined, as a treatment option for normotensive vs. hypertensive CKD, is discussed.

## 1. Introduction

Chronic kidney disease (CKD) is a global health problem that is associated with an age-related impairment of renal function, which can be exacerbated by several risk factors such as diabetes, hypertension, heart disease, and a family history of kidney failure [1]. Kidney fibrosis (or scarring that results from a failed wound-healing response to kidney damage) is the final common manifestation of CKD regardless of etiology and is the key contributor to the destruction of the kidney parenchyma that leads to end-stage renal failure (ESRD), a debilitating condition that requires dialysis or kidney transplantation as a final resort [2]. Current treatments for CKD mainly provide symptomatic management of disease progression but do not effectively treat it [3]. In this regard, bone-marrow-derived mesenchymal stromal cells (BM-MSCs) have been identified as constituting a promising treatment for CKD, owing to their immunoregulatory properties and secretion of trophic factors that mediate anti-apoptotic, mitogenic, angiogenic, anti-oxidative, and matrix-remodelling effects in the context of tissue repair [4]. However, despite the promising safety profile and renoprotective actions of BM-MSCs that have been identified from various preclinical and clinical studies, it has emerged that the poor microenvironment of chronically damaged kidneys, characterized by chronic inflammation, fibrosis, and the build-up of uremic toxins, might significantly hinder the survival and therapeutic efficacy of exogenously administered BM-MSCs [5].This, in turn, may potentially explain the lack of consistency in efficacy that these BM-MSCs have shown in clinical trials. Thus, to improve the efficacy of these cell-based therapies, many studies have sought to empower BM-MSCs by hypoxic pre-conditioning, genetic engineering, or pre-treating cells with cytokines, pharmacological drugs, or chemical agents to enhance the survival, differentiation, and regeneration potential of BM-MSCs [6,7]. This review focuses more on recent evidence that has emerged on a novel approach to reduce the fibrotic environment into which BM-MSCs can be administered into, by combining BM-MSCs with an anti-fibrotic agent, serelaxin. To enable an understanding of why this approach was developed, the review begins with an overview of the pathophysiology of CKD, with a particular emphasis on hypertension as a risk factor, and fibrosis as a hallmark of disease progression. It then summarizes key findings from various preclinical and clinical studies that have employed BM-MSCs as a therapeutic option for CKDs. After briefly outlining some of the previous strategies that have been utilized to enhance the therapeutic potential of BM-MSCs, the review concludes by discussing recent findings that have demonstrated the enhanced anti-fibrotic efficacy and renoprotection offered by the combined effects of BM-MSCs and serelaxin, and that highlight the therapeutic application of this novel combination strategy as a novel treatment option for CKD.

## 2. Chronic Kidney Disease

Chronic kidney disease (CKD) is defined as kidney damage that occurs over a long-term (greater than 3-month) period, resulting in the gradual decline of kidney function that is clinically manifested as a reduction in estimated glomerular filtration rate (eGFR; <60 mL/min/1.73 m^2^) and proteinuria (measured by an albumin-to-creatinine ratio of >30 mg/g) [8]. A grading system has been established that separates CKD patients into five categories (G1, G2, G3a, G3b, G4 and G5) based on their GFR, with each subsequent category representing a stage of worsened renal function, with G5, the most debilitating stage, defined as end-stage kidney disease (ESKD). Furthermore, a subcategory associated with albumin-to-creatinine ratio (ACR) is also incorporated into the current CKD classification (Table 1). An increase in the G- and/or A-stage is correlated with an increased risk of comorbidities and kidney failure [8].

CKD imposes a significant socioeconomic burden. The estimated prevalence for stage 3–5 CKD patients is ~10.6% [9]. With a growing ageing population and increasing prevalence of diabetes and hypertension (which are the three major risk factors for CKD) [10], the incidence of CKD is expected to increase in the coming years. In Australia alone, the total health expenditure spent on CKD was approximately 4.1 billion in 2012, and the cumulative cost attributed to all cases of end-stage renal disease (ESRD) was estimated at $11.3 billion–$12.3 billion dollars from 2009 to 2020 [11]. Moreover, CKD carries a significant risk of cardiovascular morbidity, premature mortality, and/or decreased quality of life [9]. The estimated prevalence of heart failure in CKD patients is 25% [12], which progressively increases with a more advanced disease stage and can even reach ~65–70% in ESKD patients [12].

**Table 1:** Classification of CKD using GFR and ACR categories.

GFR and ACR Categories and Risk of Adverse Outcomes			Kidney Damage Stage: ACR Categories (mg/g)		
			<30 mg/g	30–300 mg/g	>300 mg/g
			A1	A2	A3
**Kidney Function Stage:GFR (mL/min/1.73m^2^**)	≥90	Stage G1	LR	MR	HR
	60–89	Stage G2	LR	MR	HR
	45–59	Stage G3a	MR	HR	VHR
	30–44	Stage G3b	HR	VHR	VHR
	15–29	Stage G4	VHR	VHR	VHR
	<15	Stage G5	VHR	VHR	VHR

The five stages of CKD, from the mildest form (shaded in green) to the most severe form (shaded in red). Abbreviations: LR, low risk; MR, moderate risk; HR, high risk; VHR, very high risk. Adapted from [13].

Current therapeutic options for patients with CKD are limited. The management of CKD is mostly symptomatic (alleviating the symptoms or preventing the development of the complications associated with CKD, by methods such as reducing blood pressure (BP) or glucose levels and/or controlling blood cholesterol to reduce the risk of heart disease [14]). However, none of the treatments effectively halt the progression of CKD to ESKD, leaving a high demand for renal replacement therapy [14]. The grave prospective morbidity and mortality, enormous healthcare expenditure spent on CKD, and lack of effective treatments underscore the need for developing novel interventions to prevent its progression to ESKD and the socioeconomic burden associated with dialysis and/or organ transplantation.

### 2.1. Pathological Events Underlying CKD

#### 2.1.1. Hypertension

Hypertension is the second leading contributor to CKD [15]. Clinical symptoms of hypertensive kidney damage (hypertensive nephropathy) are often observed in patients with 10–15 years of persistent hypertension [16]. There are three major pathological determinants of hypertensive nephropathy: (1) systemic BP load; (2) transmission of systemic BP load to the renal vasculature; and (3) susceptibility of nephrons and local renal tissues to any given degree of increased mechanical load [17]. The renal microvasculature is normally protected by an autoregulatory system, which triggers vasoconstriction of the pre-glomerular vasculature in response to a temporal and moderate increase in systemic BP load, such that renal blood flow and glomerular hydrostatic pressure are kept relatively constant [17,18,19]. The autoregulatory BP threshold might differ among individuals due to their genetic or acquired differences in intrinsic renal structures. For instance, evidence supports that any significant pre-glomerular vasodilation (observed in uninephrectomized or early type 1 diabetic patients) or renal mass reduction significantly compromises the ability of the autoregulatory system to prevent the direct transmission of elevated BP to the renal vasculature [19]. Consequently, these patients might exhibit a more advanced stage of renal disease even with moderate hypertension due to their lower autoregulatory threshold [17]. BP elevations that exceed the autoregulatory range induce disruptive injury to the nephrons and local vasculature, which further enhances the susceptibility to renal injury by any given degree of increased BP load, thereby initiating a vicious cycle of hypertensive nephropathy [17]. Therefore, reducing BP to the normotensive range (<120/80 mmHg) in CKD patients, despite being a challenge, may represent a primary clinical strategy to halt this vicious cycle.

#### 2.1.2. Renal Fibrosis

**Figure 1:**
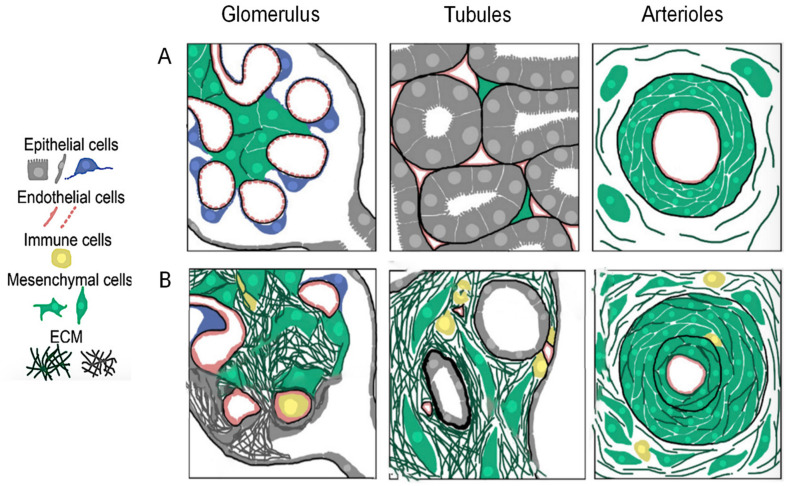
Schematic diagram showing the structural transition from (**A**) healthy to (**B**) fibrotic kidneys. The continued insult to the tissue leads to prolonged infiltration of immune cells and persistent activation and differentiation of myofibroblasts (mesenchymal cells), resulting in excessive collagen deposition within the glomerulus, renal tubules, and vasculature, which disrupts normal kidney architecture. These structural abnormalities drive progressive cell death and irreversible loss of kidney functions. Figure adapted from [14].

Fibrosis is characterized by an excessive production of ECM components, primarily collagen, due to a maladaptive wound-healing response to chronic or severe tissue injury and results from the failure of organs to effectively repair and regenerate [20]. Renal fibrosis refers to tubulointerstitial fibrosis, glomerulosclerosis, or vascular fibrosis and is the final common manifestation of a wide variety of CKDs, irrespective of their etiology [14]. The underlying cellular events are complicated and involve interactions of multiple kidney resident cells as well as infiltrating cells, tubular epithelial-to-mesenchymal transition (EMT), monocyte/macrophage and T cell infiltration (and cell apoptosis), and interstitial fibroblast and glomerular mesangial cell differentiation into activated smooth muscle-containing myofibroblasts. The infiltration and differentiation (activation) of these cells are stimulated by a variety of cytokines and growth factors derived from adjacent epithelial cells, endothelial cells, and immune cells or from fibroblasts themselves [14] (Figure 1). In the aftermath of prolonged renal injury, pro-inflammatory cells (such as neutrophils, monocytes, and T cells) that infiltrate and are activated at damaged sites release excessive pro-inflammatory cytokines (such as tumor-necrosis factor (TNF)-α, interleukin (IL)-1β and IL-6, amongst other) to clear bacteria and cell debris from the wound site. These cells also release a number of pro-fibrotic factors, most notably angiotensin (Ang) II and transforming growth factor (TGF)-β1, that upon binding to their angiotensin type 1 receptor (AT_1_R) and TGF-β1 type I and II receptors, respectively, drive the overactivation of myofibroblasts and continuous production of highly crosslinked ECM, which in turn traps more inflammatory cells within damaged tissues to exacerbate the release of the above-mentioned mediators [2].

#### 2.1.3. Angiotensin II and TGF-β1

**Figure 2:**
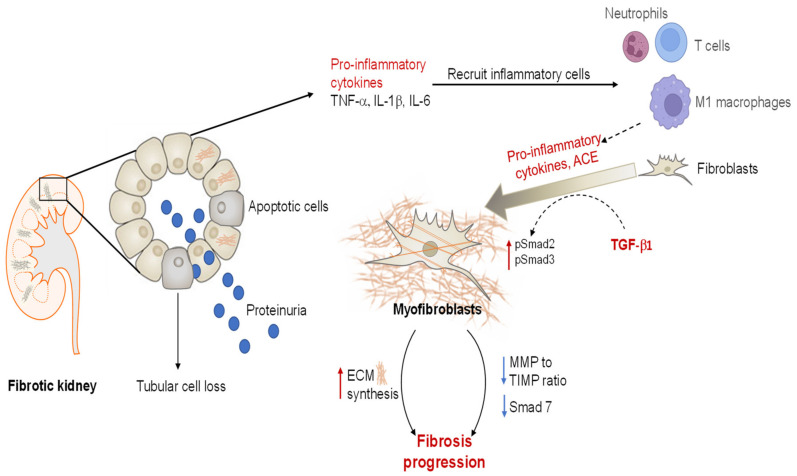
Fibroblast-to-myofibroblast transition during fibrogenesis, a key event in renal fibrogenesis. Following tissue injury, resident macrophages secrete cytokines such as TNF-α, IL-1β and IL-6 to promote infiltration of more immune cells (such as macrophages and neutrophils). Macrophages also release TGF-β1 and ACE, which convert Ang I to Ang II, both of which are pro-fibrotic factors that directly promote fibroblast-to-myofibroblast transformation, via a Smad-dependent pathway. Furthermore, the balance between the matrix-degrading MMPs and their inhibitors, TIMPs, is dysregulated, leading to impaired ECM turnover. In summary, the excessive matrix deposition observed in fibrotic kidneys results from a combination of overproduction of ECM proteins and defective ECM degradation.

The intrarenal renin–angiotensin–aldosterone system (RAAS) is found to be consistently upregulated in CKD and ESKD patients to maintain BP control in these injured people. As a central component of the RAAS, Ang II plays a key role in hemodynamic regulation and renal pathology, with its over-expression well-correlated with a loss of kidney function [21]. Ang II is generated via proteolytic cleavage of angiotensin I (Ang I) by the angiotensin-converting enzyme (ACE), produced by infiltrating macrophages and fibroblasts [22,23]. Hemodynamically, Ang II elevates trans-glomerular pressure directly by inducing the constriction of efferent arterioles, and indirectly by inducing TGF-β1-mediated dysregulation of the above-mentioned renal auto-regulatory system [24]. Ang II also stimulates inflammation, hypertrophy, cell proliferation, and reactive oxygen species during wound healing, and acts as a potent stimulant of the proliferation and differentiation of resident renal fibroblasts, either via the AT_1_R, which promotes the direct effects of Ang II, or indirectly via its downstream activation of TGF-β1 remodelling [21,25,26]. TGF-β1 (mainly released from macrophages and regulatory T cells (Tregs)) is recognized as the most potent inducer of myofibroblast differentiation [2]. The profibrotic effects of TGF-β1 are mainly achieved through its ability to promote the transition of fibroblasts into activated myofibroblasts, enhancing the myofibroblast-mediated synthesis of highly crosslinked ECM within the kidney interstitium and basement membrane [2,27], and interrupting the balance between ECM-degrading matrix metalloproteinases (MMPs) and their natural inhibitors, the tissue inhibitors of metalloproteinases (TIMPs) [2,27,28] (Figure 2). TGF-β1 binding and activation of its tyrosine kinase type I and II receptors trigger the phosphorylation of downstream intracellular mediators, Smad2 (pSmad2) and Smad3 (pSmad3), before they bind to a common partner, Smad4 [27]. This trimeric complex subsequently translocates from the cytoplasm to the nucleus, where it localizes to Smad binding elements (SBE) in the enhancer and promoter regions of the target genes that stimulate collagen and fibronectin production as well as the differentiation of fibroblasts to myofibroblasts [27]. The presence of co-repressors of Smad transcription (e.g., SnoN, Ski, and TGIF) and Smad7 (an inhibitory Smad) acts as a safe-guard to prevent overactivation of the remodelling pathway in a negative feedback loop. However, in tissues with fibrotic lesions, inhibitors of the Smad2/3/4 activity are usually progressively abrogated, which further promotes and amplifies TGF-β1-mediated fibrogenesis [27]. Meanwhile, an upregulation of TGF-β1 can induce a reduction in MMP-to-TIMP ratio, which leads to dysregulated collagen turnover [2]. Taken together, the formation of fibrosis is attributed to an aberrantly upregulated accumulation of ECM, accompanied by inefficient ECM degradation (Figure 2) [2]. Additionally, the kidneys have a limited capacity for tissue repair and remodelling, particularly when subjected to chronic insults [4]. These structural abnormalities disrupt normal cellular functionality, driving the irreversible death of nephrons that contributes to a progressive loss of kidney function.

Current first-line treatments that were developed to inhibit intrarenal RAAS activity, including ACE inhibitors (ACEi) and angiotensin receptor blockers (ARBs) as monotherapies, do not produce the level of renal protection that was expected by blocking a pathway that is intimately linked to the pathogenesis of CKD depicted above. Instead, they have only shown modest effects in reducing established fibrosis (through their well-established anti-hypertensive and anti-inflammatory effects), and as a result of this have only improved survival rates of patients with CKD-induced ESKD by a few months [3,29]. Furthermore, the Ongoing Telmisartan Alone and in combination with Ramipril Global Endpoint Trial (ONTARGET; ClinicalTrials.gov ID: NCT00153101) showed disappointing results regarding the dual inhibition of RAAS with ramipril (an ACEi) and telmisartan (an ARB) [30]. The combination treatment induced more adverse events than either treatment alone, including hyperkalaemia, hypotension, and even acute kidney injury, which led to premature termination of the trial. For patients reaching ESKD, dialysis and kidney transplant are often the only viable therapeutic options [31]. This underscores the need for developing novel interventions that can effectively prevent CKD progression to ESKD and the costs associated with dialysis and organ transplantation.

## 3. Mesenchymal-Stem-Cell-Based Therapy

The development of novel therapeutic strategies for CKD-induced ESKD needs to incorporate an understanding of the process of endogenous repair and regeneration adopted by the kidney after injury. The formation of nephrons usually ceases at birth in the kidneys of mammals [32]. Conversely, adult kidneys have some capacity of endogenous cellular replacement and remodelling that restores renal structure and function following acute kidney damage, which is predominantly achieved by dedifferentiation and migration of the surviving tubular epithelial cells from the site of apoptosis and cell detachment post-injury, where they proliferate and re-differentiate into functional tubular epithelial cells [33,34,35]. However, previous studies have suggested a relatively small con-tribution of Foxd1 stromal cells and other resident stem cells in the regeneration and re-modelling of newly viable tubular cells [34,36,37]. Over the last couple of decades, the field of regenerative medicine has emerged as a novel promising strategy to modulate the disease progression of CKD. It has become clear that the activation of endogenous repair mechanisms can be aided by various types of progenitor cells, amongst which mesenchymal stem/stromal cell (MSC)-based therapies have attracted the most considerable attention.

MSCs are self-renewing adult stem cells of mesodermal origin and can be differentiated into cells of mesodermal lineage including osteocytes, adipocytes, and chondrocytes [4,38]. According to the minimal criteria set by the International Society for Cellular Therapy (ISCT), MSCs are plastic-adherent in standard tissue culture conditions, exhibit fibroblast-like morphology, and are characterized by their surface expression of cluster of differentiation (CD)73, CD90, and CD105 antigens [39]. MSCs lack expression of hematopoietic markers (CD45, CD34, and CD14), co-stimulatory molecules (CD40, CD80 and CD86), and major histocompatibility complex class II (MHC II), rendering these cells immune-privileged and hence making allogenic transplantation of MSCs a safe and viable therapeutic option [4,40]. Furthermore, unlike other progenitor cells such as embryonic stem cells and induced pluripotent stem cells, MSCs do not cause teratoma formation [41]. The most investigated form of MSCs, bone-marrow-derived MSCs (BM-MSCs), are easily obtained from young, healthy donors, have low immunogenicity, and can easily be manufactured at a large scale. Hence, BM-MSCs are the focus of this review and will be discussed hereafter.

### 3.1. Mechanisms Underlying the Reparative Effects of BM-MCSs

**Figure 3:**
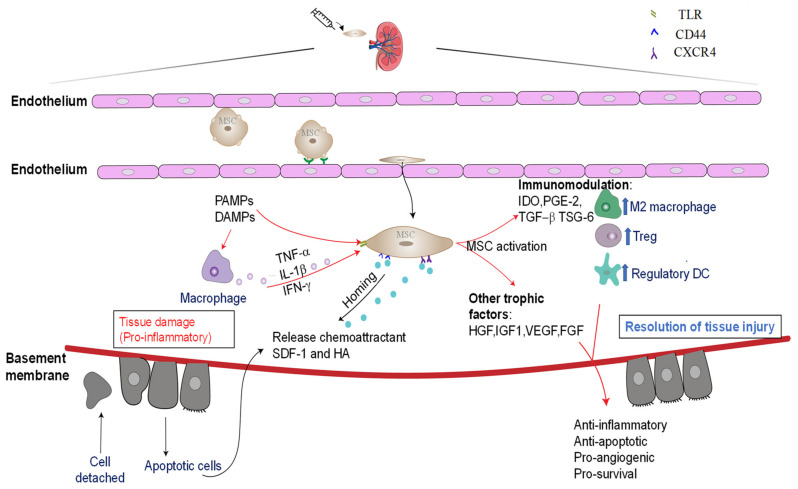
Renoprotective effects of BM-MSCs mediated via immunomodulation and secretion of tropic factors. Following infiltration into the injured tissue, BM-MSCs interact with various adhesion molecules lining the endothelium to ensure their localization to the damaged site. The site of primary insult releases chemoattractant SDF-1 and HA, which bind to CXCR4 and CD44, respectively, on the surfaces of BM-MSCs to enhance their homing. The local microenvironment contains numerous pro-inflammatory factors released by the damage area (such as IL-1β and TNF-α), which along with toll-like receptor activation, is critical for the priming and activation of BM-MSCs. Once activated, BM-MSCs release a wide range of trophic factors and mediate immunomodulatory effect to resolve tissue inflammation and promote structural and functional impair.

Despite exogenously delivered BM-MSCs being found to repopulate the injured renal tubular epithelium via direct engraftment and trans-differentiation [42], recent evidence has shown that the renoprotective effects of BM-MSCs are primarily mediated in a paracrine manner, via the secretion of trophic factors and by immunomodulation [40,43]. BM-MSCs have numerous surface chemokine receptors, including C-C chemokine receptor (CCR)1, CCR2, CCR2, CCR4, CCR7, C-X-C chemokine receptor (CXCR)-4, CXCR-5, CXCR6, and hyaluronan receptor CD44, that may interact with the chemokines (such as stromal cell-derived factor 1 (SDF1) and hyaluronic acid (HA)) that are released under pathological conditions and assist in their migration to sites of inflammation [4,44,45]. Once at the site of injury, BM-MSCs release a broad repertoire of trophic and regulatory molecules, including growth factors, proteinases, hormones, cytokines, and chemokines, which are collectively known as the MSC secretome [46]. It is now understood that the therapeutic effects of BM-MSCs are primarily mediated via the release of these soluble factors, which interact closely with the local microenvironment to modulate inflammatory, fibrogenic, and hypoxic responses as a way to promote tissue repair and regeneration [47]. The anti-apoptotic and mitogenic growth factors secreted by BM-MSCs, such as hepatocyte growth factor (HGF), insulin-like growth factor (IGF-1), vascular endothelial growth factor (VEGF), fibroblast growth factor (FGF)-II, basic nerve growth factors, epidermal growth factors (EGF), and platelet derived growth factors (PDGF), have all been found to contribute to cellular proliferation at injured sites [48]. BM-MSCs also release angiogenic (such as VEGF, angiopoietin-1, and monocyte chemoattractant protein (MCP)-1) and chemotactic factors that further aid recovery of the damaged tissue by promoting vasculogenesis and angiogenesis [49]. These factors also mediate ECM remodelling by secreting ECM and cell adhesion proteins and modulate the activities of matrix-degrading MMPs [50] (Figure 3).

BM-MSCs have well-documented immunomodulatory properties, which means they have the ability to modulate the functional characteristics and cytokine profiles of the major immune cell subsets associated with both innate and adaptive immune responses, including natural killer cells, macrophages, dendritic cells, T cells, and B cells [40]. The relative contribution of each immunosuppressive mechanism may vary, as the specific immune cell types that are primarily activated or suppressed by BM-MSCs differ depending on the etiology of the disease studied [51]. In terms of innate immunity, studies have shown that BM-MSCs are able to suppress the expression of toll-like receptor 3 (TLR3) and TLR9 and attenuate the migration of dendritic cells (DCs) via mitigating the expression of lymph node homing chemokine receptor CCR7 [52]. BM-MSCs can also inhibit the maturation of DCs by down-regulating DC maturation markers including major histocompatibility complex (MHC) class II, CD40, CD80, and CD86 [53], which directly hinders the local antigen priming of T cells [52]. Notably, BM-MSC secretion of IL-6, macrophage colony stimulating factor (M-CSF), PGE2, and IL-10 have all been implicated in the induction of tolerogenic DCs [54,55].

Given that macrophages play a central role in inflammation, the mechanisms underlying the MSC–macrophage interaction have also been intensively investigated [4,56]. In response to different signals, macrophages can be polarized into either a classical M1 phenotype or an alternatively activated M2 subtype [51]. M1 macrophages exhibit potent antimicrobial properties and are responsible for the promotion of Th1 response (characterized by an elevated production of pro-inflammatory cytokines, including IL-1β, IL-6, and TNF-α) [57]. Conversely, M2 macrophages secrete fewer pro-inflammatory cytokines (characterized by high levels of IL-10 production and low levels of IL-12, TNF-α, and IL-1α), promote Th2-type immune responses, and display a highly phagocytic phenotype [57,58]. Therefore, the alternatively activated M2 macrophages are believed to be involved in the resolution of inflammation. Substantial evidence has indicated that BM-MSCs can polarize the pro-inflammatory M1 macrophage to a regulatory M2 subtype, as M1 macrophages that were co-cultured with BM-MSCs for 72 h had elevated expression of CD206 (a marker of M2 macrophages) with concomitant high-level expression of anti-inflammatory cytokine IL-10 and low levels of pro-inflammatory cytokines including IL-12, TNF-α, and IL-1α [58,59]. The potential mechanisms by which BM-MSCs orchestrate macrophage polarization have been elegantly elucidated in a murine model of sepsis [60]. Briefly, lipopolysaccharide (LPS) and TNF-α enriched in the inflammatory microenvironment activate TLR4 and TNF receptor 1 (TNFR1) on BM-MSCs to induce NF-κB signalling that upregulates cyclooxygenase (COX)-2 expression. This in turn, leads to an increased release of PGE2 from BM-MSCs, which reprograms macrophages by binding to their surface-expressed prostaglandin receptors, EP2 and EP4, to enhance IL-10 production [60]. Later, Choi and colleagues suggested another potential approach by which BM-MSCs re-treat macrophage phenotype in a zymosan (a TLR-2 agonist)-induced peritonitis model, where they found that the activation of NF-κB signalling from macrophages triggered by zymosan led to the BM-MSC production of TNF-α and TNF-α-stimulated gene-6 (TSG-6), which then interacted with CD44 on the surface of macrophages to initiate a negative feedback loop that inhibited NFκB signalling and associated inflammatory responses [59]. The same mechanism could also be used to explain BM-MSC-mediated inhibition of NF-κB activity in T helper cells and DCs in models of diabetes [61] and corneal transplant rejection [62]. Nonetheless, the BM-MSC–macrophage interaction plays an essential role in the immunomodulation of BM-MSCs, and the mechanism of actions deployed by BM-MSCs seems to be largely dependent on the micro-environmental cues at the site of BM-MSC activation. Regarding the adaptive immune response, BM-MSCs have been found to suppress interferon (IFN)-γ production and proliferation of CD4^+^ and CD8^+^T lymphocytes [63], impair the cytotoxic function of naïve and memory T cells, and promote CD4^+^CD25^+^ regulatory T cell (Treg) production through modulating immunosuppressing factors such as PGE2, IL-10, indoleamine-2,3-dioxygenase (IDO), and nitric oxide (NO) [64].

Notably, emerging evidence has suggested that the inflammatory and hypoxic responses in the local environment are responsible for enabling the immunosuppressive effect of BM-MSCs, as the above-mentioned soluble factors are minimally expressed in resting BM-MSCs [51]. In particular, pro-inflammatory cytokines such as IFN-γ, TNF-α, or IL-1β are enriched at the site of injury. Furthermore, damage-associated molecular patterns (DAMPs), pathogen-associated molecular patterns (PAMPs), as well as TLR-3 and TLR-4 signalling have all been implicated in priming BM-MSCs to exert their immunosuppressive effects (Figure 3). Taken together, through secretion of soluble trophic factors and via immunomodulation, BM-MSCs shift the overall immunity from pro-inflammatory Th1/Th17-driven immune responses towards a more anti-inflammatory Th2/Treg profile, thereby discontinuing the prolonged inflammation phase and stimulating the progression of cell proliferation and tissue remodelling, which are critical for the functional recovery of chronically injured organs.

### 3.2. Preclinical Evidence for BM-MSCs as a Treatment Option for Chronic Models of Kidney Injury

Several in vivo studies have confirmed that BM-MSCs prevent the progression of or even reverse certain stages of experimental CKDs, as indexed by significant improvements in clinically used functional markers, including a reduction in plasma creatinine, plasma urea, urinary protein levels, and/or an increase in GFR (Table 2). These functional alterations are partly explained by the anti-fibrotic effects of BM-MSCs, which have been demonstrated in several normotensive [65] and hypertensive [66] models of CKD. Notably, the anti-fibrotic effects of BM-MSCs are likely to overlap with their anti-inflammatory properties, and their modes of anti-fibrotic actions seem to fall under four main categories, two of which are (i) immune modulation and (ii) inhibition of TGF-β1 activity. In particular, BM-MSC transplantation reduces TGF-β expression and the downstream phosphorylation of intracellular Smad2 (upon TGF-β1 binding to its receptors), which in turn suppresses the number of α-SMA-positive cells [67,68,69]. This results in reduced levels of myofibroblast proliferation and differentiation, and myofibroblast-induced ECM production; and epithelial-to-mesenchymal transition (EMT), which is a known contributor to renal fibrosis via phenotypic changes of the tubular epithelium into myofibroblasts [65,69]. Furthermore, a decreased expression of connective tissue growth factor (CTGF) has also been reported following BM-MSC administration [68]. The TGF-β1-inhibitory roles of BM-MSCs might be associated with the cell-released hepatocyte growth factor (HGF) and TNF-stimulated gene 6 (TSG-6) [50,70]. The anti-fibrotic properties of BM-MSCs can also be mediated by the two remaining categories, namely their ability to (iii) inhibit oxidative stress and (iv) stimulate matrix remodelling (via the promotion of MMP-induced collagen degradation and/or the downregulation of TIMPs) (Table 2). Conversely, Huuskes et al. [71] reported a lack of BM-MSC-mediated anti-fibrotic effects in mice subjected to one week of unilateral ureteric obstruction (UUO)-induced tubulointerstitial kidney disease, in which BM-MSCs alone failed to prevent the progression of tubulointerstitial and total renal collagen accumulation by 7 days post-injury. The authors proposed that the fibrotic microenvironment of the obstructed kidney likely compromised the survival and efficacy of the exogenously-administered BM-MSCs, which led to the hypothesis that combining BM-MSCs with an agent that has more efficacious anti-fibrotic properties may enhance BM-MSC-mediated renoprotection and repair in CKD settings [71]. It is also probable that the anti-fibrotic (and therapeutic) efficacy of BM-MSCs may vary depending on the timing/severity of when they are exogenously administered to injured organs, route of administration, and/or the type of disease etiology to which they are applied.

Additionally, BM-MSCs have exerted anti-hypertensive effects in experimental models of hypertensive CKDs, which is of significant clinical importance, as systemic hypertension is one of the most prominent contributors to both the initiation and progression of kidney damage. Despite the specific mechanisms involved being poorly understood, studies to date have suggested that BM-MSCs act through several modes of action to induce their anti-hypertensive effects, including an ability to inhibit (i) NLRP3 inflammasome assembly and activity [72]; (ii) immune cell infiltration (e.g., macrophages and T cells); and (iii) pro-hypertensive components of the RAAS; whilst (iv) mitigating sympathetic nerve activation after localizing to the central nervous system. For example, in a 2 kidney–1 clip (2K1C) model of renovascular hypertension, BM-MSCs blunted the expression of intrarenal RAS components including angiotensinogen, renin, ACE, and AT_1_R expression in the medulla of the clipped kidney and prevented further increases in systolic BP (SBP) [73,74]. Interestingly, the same group also detected a trace amount of BM-MSCs (~2%) in the medulla oblongata at one week post-transplantation [73]. As the medulla oblongata plays a critical role in regulating autonomic functions such as BP and heart rate, the previous findings indicated that BM-MSCs may cross the blood–brain barrier and localize to the CNS after intravenous (iv) delivery, where they mitigate sympathetic hyperactivation, a major contributor to the initiation and maintenance of hypertension. BM-MSCs also interact with and down-regulate AT_1_R expression in the paraventricular nucleus (PVN; in hypothalamus) and rostral ventrolateral medulla (RVLM; receiving axonal projections from paraventricular nucleus) [75,76]. These findings collectively indicated that the anti-hypertensive actions of BM-MSCs were partly mediated by their central roles. Activation of immune cells and NLRP3 inflammasomes have both been implicated in many chronic inflammatory conditions, including hypertension and the associated kidney injury [77]. BM-MSCs were found to suppress the high-salt-induced assembly and activation of NLRP3 inflammasomes (including NLRP3 and caspase 1) and the downstream production of IL-1β in Dahl salt-sensitive rats and 1K/DOCA/salt-injured mice and inhibit innate (macrophages) and adaptive immune (T cells) components in a murine model of fibrotic nephropathy induced by unilateral ureteral obstruction (UUO) [71] and 1K/DOCA/salt-induced hypertensive mice [72,78]. Therefore, the ability of BM-MSCs to inhibit inflammasome activity and immune cell infiltration may also explain the anti-hypertensive and anti-inflammatory effects of these stem-cell-based therapies.

Taken together, preclinical evidence has collectively revealed the renoprotective role of BM-MSCs in CKD settings, mainly by downregulating major signalling mediators responsible for disease progression (e.g., inflammation, oxidative stress, and fibrosis). More importantly, BM-MSC-based therapies also suppress BP load via several mechanisms in hypertensive models, indicating their therapeutic potential to complement current anti-hypertensive treatments for normotensive and even hypertensive patients with renal dysfunction (Table 2).

**Table 2:** Examples of preclinical studies evaluating the therapeutic effects of BM-MSCs in models of chronic kidney diseases due to various etiologies.

Etiology	In Vivo Models	MSC Number and Source	Routes of Delivery	Main Outcomes	Reference(s)
**Hypertension**	2K1C induced renovascular hypertension	1 × 10^6^ rat BM-MSCs	Subcapsular injection	↓ SBP ↓ Renin, ACE, and AT_1_R expression ↓ Renal Na+/K+ ATPase activity ↓ TGF-β1 and fibrosis ↓ Proteinuria ↑ AT_2_R expression ↑ Kidney morphology	[79]
	2K1C induced renovascular hypertension	2 × 10^5^ rat BM-MSCs	iv injection	↓ SBP ↓ Sympathetic hyperactivity ↓ Angiotensinogen, ACE, and AT_1_R levels ↓ Fibrosis, inflammation, and proteinuria	[73]
	2K1C induced renovascular hypertension	1 × 10^6^ rat BM-MSCs	iv injection	↓ Inflammation and oxidative stress ↓ Morphological and ultrastructural abnormalities ↓ Serum urea and creatinine	[80]
	High-salt diet (8% NaCI)	5 × 10^6^ rat BM-MSCs	Intra-renal infusion	↓ SBP ↓ Inflammasome activation ↓ Hypertensive kidney damage	[81]
	5/6 subtotal nephrectomy	2 × 10^5^ rat BM-MSCs	iv injection	↓ Fibrosis indices (collagen I, vimentin, TGF-β, α-SMA) ↓ Inflammation	[66]
	5/6 subtotal nephrectomy	2 × 10^5^ rat BM-MSCs	Subcapsular injection	↓ SBP ↑ Renal function (↓ Albuminuria, serum creatinine, GS)	[82]
	1K/DOCA/salt	1 × 10^6^ human BM-MSCs	iv injection	↓ SBP ↑ Renal function (↓ Proteinuria,↑creatinine clearance) and morphology ↓ Inflammation and fibrosis	[72,78]
**Obstructive** **nephropathy**	Unilateral ureteric obstruction (UUO)	1 × 10^6^ human BM-MSCs	iv injection	↓ Inflammation No anti-fibrotic effect	[71]
**Nephrotoxicity**	Cisplatin-induced chronic kidney damage	3 × 10^6^ rat BM-MSCs	iv injection	↓ Creatinine and urea ↓ Inflammation and fibrosis ↑ Hepatocyte growth factor (protective for renal epithelial cells)	[65]

### 3.3. BM-MSC Therapy in Clinical Trials for Managing CKD

According to clinicaltrials.gov, there are currently 45 ongoing or completed clinical trials evaluating the safety and efficacy of mesenchymal stem cells in treating CKD due to various etiologies. Of these, only studies using BM-MSCs are discussed herein (see Table 3).

In a phase I, single-arm, open-label clinical trial (ClinicalTrials.gov ID:NCT02195323), seven eligible participants with CKDs due to hypertension, nephrotic syndrome, or unknown causes received a single dose of 2 × 10^6^ autologous BM-MSCs and were followed for 18 months post-cell-transplantation [83]. The primary aim of this study was to investigate the safety of BM-MSC administration in CKD patients. Although no remarkable changes were observed in eGFR or serum creatinine in BM-MSC-treated patients compared to the baseline levels measured, the autologous BM-MSCs demonstrated a promising safety profile, as no cell-related adverse events were reported during the 18-month follow-up period. Similar findings were obtained in another phase I exploratory study in six patients (ClinicalTrials.gov ID: NCT02166489) with advanced autosomal dominant polycystic kidney disease (ADPKD) (eGFR: 25–32 mL/min per 1.73 m^2^), in which the administration of the autologous BM-MSCs was not associated with any adverse events during a 12-month follow-up period [84]. Unfortunately, the findings from these two studies both failed to provide evidence regarding the renoprotective efficacy of BM-MSCs. However, the power of these preliminary data was quite limited due to the small sample size incorporated and lack of an appropriate control group.

In another larger-scale, dose-escalating, randomized, placebo-control pilot study (ClinicalTrials.gov ID: NCT01576328), 61 patients with type 2 diabetes were randomized to receive a single intravenous injection of 0.3 × 10^6^/kg (*n* = 15), 1 × 10^6^/kg (*n* = 15), or 2 × 10^6^/kg (*n* = 15) BM-MSCs or a placebo (*n* = 16). No infusion-associated acute adverse events, treatment-induced serious adverse events, or hypoglycemia was reported after 12-weeks of BM-MSC treatment [85]. Given the well-established safety profile of BM-MSCs mentioned above, the same group conducted a multicenter, randomized, double-blinded, dose-escalating placebo-controlled trial (ClinicalTrials.gov ID: NCT01843387) in which 30 participants with moderate to severe diabetic nephropathy were randomized to receive a single intravenous infusion of allogenic BM-MSCs (150 × 10^6^ cells or 300 × 10^6^ cells) or placebo and followed for 60 weeks post-treatment. No serious adverse events associated with infusion or deemed treatment-related or development of specific anti-HLA antibodies against donor cells was reported. Efficacy analysis revealed only a trend towards improving eGFR 12 weeks after BM-MSC infusion, which was maintained over the 60-week study period. Neither of the two dosages tested induced significant improvements in the end-point parameters measured, including urinary albumin, albumin–creatinine or protein–creatinine ratio, creatinine clearance, lipid profile, and glycated hemoglobin (HbA1c) level at week 12 [86], demonstrating a lack of efficacy of naïve BM-MSCs in the management of diabetic nephropathy.

Systemic lupus erythematous is an autoimmune disorder mediated by auto-reactive T and B lymphocytes, which can result in life-threatening situations such as lupus nephritis, in which up to 30% of patients eventually develop ESRD [87]. Despite the advances in immunosuppressive therapies, treatment-associated toxicity and rates of relapse are high; thus, a novel treatment with lower toxicity is warranted [87]. Previous clinical studies have reported on the effect of allogeneic BM-MSCs in the management of lupus nephritis. In a single-arm, open-labelled pilot (phase I/IIa) study (ClinicalTrials.gov ID: NCT00698191), 15 patients with resistant systemic lupus erythematous (SLE) received a single intravenous infusion of 1 × 10^6^/kg allogenic BM-MSCs, continued treatment with steroids at the time of infusion, and maintenance treatment 1 month later including prednisone (5–10 mg/day) and cyclophosphamide (0.4–0.6 g/2–3 months) and were followed up for 17.2 ± 9.5 months [88]. No serious adverse events were reported in any of the 15 patients. Allogenic BM-MSCs remarkably improved SLE Disease Activity Index score (SLEDAI; a validated measure of disease activity) and suppressed serum levels of autoreactive antibodies. An overall significant reduction in proteinuria was observed in all follow-up visits over the 12-month post-transplantation study period in 13 patients, despite two patients having a relapse of proteinuria [88]. It was proposed that the aforementioned MSC-mediated renoprotective effects may have been due to MSC-induced immunosuppressive effects via expansion of circulating CD4^+^Foxp3^+^Tregs, which occurred at 1 week post-treatment and were maintained until 6 months post-treatment. However, the exact mechanisms involved remained ill-defined in this study.

**Table 3:** Examples of clinical studies investigating BM-MSCs as a treatment for CKD.

Clinical Trial Number/Reference	Center	Study Details	No. of Patients	Main Outcomes
NCT02195323 [83]	Royan Institute, Tehran, Iran	iv injection of 2 × 10^6^/kg autologous BM-MSCs Phase 1	7	***Safety:*** No cell-related adverse events were reported during 18 months of follow-up ***Efficacy:*** No remarkable changes were observed in eGFR and serum creatinine in BM-MSC-treated patients compared to the baseline levels
NCT02166489 [84]	Royan Institute, Tehran, Iran	iv injection of autologous BM-MSCs, 2 × 10^6^ cells/kg Phase 1	6	***Safety:*** No MSC-related adverse events during 12 months of follow-up
NCT01576328 [85]	Mesoblast, Ltd., Melbourne, Australia	iv injection of allogenic BM-MSCs, 0.3 × 10^6^, 1 × 10^6^, or 2 × 10^6^ cells/kg Phase 1/2	61	***Safety:*** No cell-related adverse events over 12 weeks ***Efficacy:*** Clinical glycated haemoglobin target of <7% was achieved in 33.3% of participants in the 2 × 10^6^/kg group, versus none in the placebo group
NCT01843387 [86]	Mesoblast, Ltd., Melbourne, Australia Monash University, Clayton, Australia Melbourne Renal Research Group, Melbourne, Australia	iv injection of allogenous BM-MSCs, at 150 × 10^6^ or 300 × 10^6^ cells/kg Phase 1/2	30	***Safety:*** No cell-related adverse events ***Efficacy:*** A trend towards stabilizing eGFR at week 12, which was maintained until 60 weeks of follow-up
NCT00698191 [88]	Nanjing Medical University, China	iv injection of allogeneic BM-MSCs, at 1 × 10^6^/kg iv	15	***Safety:*** No cell-related adverse events ***Efficacy:*** Amelioration of disease activity, with improvement in serologic markers and renal function

In summary, results from various clinical trials to date have shown a promising safety profile and well-established immunosuppressive effects of BM-MSCs for treating various CKD conditions (Table 3). Despite this, some potential risks should be considered when using exogenously expanded BM-MSCs for clinical applications, including (1) the risk of opportunistic infections due to overimmunosuppression [89]; (2) the risk of immunogenic responses to transplanted cells [90]; (3) ectopic tissue formation [91,92]; and (4) the risk of tumorigenesis from the abnormal transformation of BM-MSCs [93]. Although no malignancies, ectopic tissue formation, severe immunogenic responses, or opportunistic infections were reported in the clinical trials cited above, a longer follow-up period post-BM-MSC delivery is warranted. Furthermore, most of the clinical trials conducted so far have only assessed the indirect application of BM-MSCs through iv administration rather than directly into the kidneys via intra-renal (ir) administration, which might dampen the efficacy of BM-MSCs within the kidney, as the majority of iv-infused cells initially migrate via circulation into the lungs [94] before reaching sites of damage. Hence, future trials comparing the safety and efficacy of direct versus indirect administration of BM-MSCs for the management of CKD are required.

**Figure 4:**
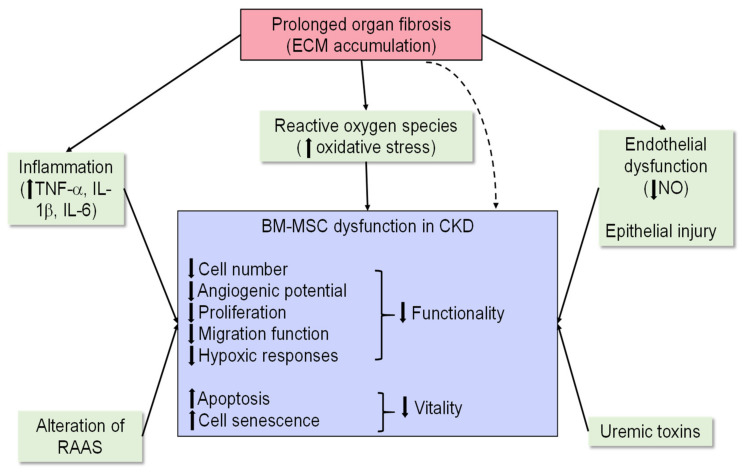
The functionality (indicated by the number of circulating stem cells and their tissue-reparative efficacy) and vitality (cellular survival and senescence) of transplanted BM-MSCs can both be impaired due to a deleterious organ environment created by prolonged organ fibrosis and other factors depicted above, which override the tissue-reparative benefits of BM-MSCs in CKD.

Furthermore, the successful clinical transition of BM-MSCs (as a treatment for CKD) is still hindered by a poor understanding of their mechanisms of action in human patients and a lack of consistent efficacy profile of these stem cells in clinical trials (for diabetic or hypertensive nephropathy) despite tremendous evidence of their renoprotective effects in preclinical studies. One major concern of BM-MSC therapy is that when exogenously administered, these cells may only have a temporary and marginal efficacy, particularly in severe injury or chronic disease settings, in which transplanted BM-MSCs have been shown to suffer from poor survival and engraftment, which directly hampers their therapeutic effects [7]. Preclinical studies have characterised the altered phenotypes of BM-MSCs in chronically injured kidneys, where they exhibited reduced proliferative capacity, expression of VEGF receptors and chemoattractant SDF-1α, and enhanced tendency towards senescence [5,95]. The main factor underlying the impaired functionality of this cell-based therapy is speculated to be the inhospitable micro-environment created by the chronically injured kidney, characterised by inflammation, increased oxidative stress production, endothelial dysfunction, and the release of uremic toxins (referring to biologically active compounds that are retained due to kidney damage). Most importantly, the establishment of fibrosis has been found to directly impair the viability and efficacy of MSC-based therapies via its suppression of the survival, proliferation, and subsequent migration of implanted BM-MSCs to the injured site, which is further exacerbated by tissue inflammation and reduced nitric oxide (NO) availability attributed to an excessive build-up of ECM/collagen deposition [5,6,96] (Figure 4). All these factors might limit the ability of the injured kidney to harvest donor cells and compromise the efficacy of MSC-based therapies in various CKDs. Taken together, these findings have somewhat suggested that naïve BM-MSCs have failed to consistently demonstrate anti-fibrotic as well as renoprotective effects in treating CKD and hence may not be effective alone to treat severe or chronic injury. Hence, pre-treatment of BM-MSCs or an adjunct therapy to BM-MSC administration might be required to enhance the therapeutic efficacy of these cells, especially in chronic disease settings.

## 4. Strategies Used to Enhance BM-MSC-Based Therapies

In light of these issues, various researchers have sought to improve the efficacy of BM-MSC-based therapies in treating severe or chronic injury. To date, emerging concepts such as (1) pre-conditioning BM-MSCs with cytokines or certain cytoprotective factors; (2) genetic modification of BM-MSCs (to over-express specific microRNAs and anti-inflammatory or angiogenic factors); (3) application of a supporting material such as hydrogels; and (4) combination therapy have all been implicated in aiding the therapeutic potential of BM-MSCs [7,47]. These strategies were designated to enhance the survival, engraftment, immunomodulatory, and paracrine effects of exogenously administered BM-MSCs. For example, pre-conditioning BM-MSCs with S-nitroso N-acetyl penicillamine (SNP; a nitric oxide donor) in vitro reduced BM-MSC apoptosis via the promotion of cytoprotective gene expression [97]. When tested in a rat model of renal ischemia–reperfusion injury, SNP enhanced the proliferation and engraftment of BM-MSCs to the ischemic kidney, induced a higher expression of pro-survival and pro-angiogenic factors, and improved kidney function to a greater extent than unmodified BM-MSCs alone [97]. Furthermore, pre-treatment with melatonin, a hormone that regulates circadian rhythms, protected BM-MSCs in CKD patients from oxidative stress and cellular senescence in vitro via PrP^C^-dependent mitochondrial functional enhancement [98]. Similarly, melatonin-treated BM-MSCs exhibited enhanced survival and angiogenic effects at engraftment sites following prolonged renal ischemia in vivo, compared with unmodified BM-MSC-administered controls [98].

The genetic engineering of BM-MSCs has been employed to enhance several properties of these cells that have directly been linked to their therapeutic outcome(s), including the (1) homing (e.g., when over-expressing insulin-like growth factor IGF-1 [99] or C-C chemokine receptor type 1 CCR1 [100]); (2) survival (via hypoxic pre-conditioning and over-expression of the survival protein Akt) [101,102]; and (3) angiogenic (when over-expressing VEGF and bFGF) [103] (4) anti-inflammatory (e.g., when over-expressing IL-18-binding protein) [104]; (5) anti-apoptotic (e.g., when over-expressing BCL-2) [105]; and (6) anti-fibrotic (e.g., when over-expressing microRNA-Let 7c) [106] effects of these cells.

Combining BM-MSCs with other agents such as atorvastatin (a lipid-lowering medication) or darbepoetin-α (DPO; an erythropoietic agent) has also been shown to induce greater functional recovery of the ischemic kidneys than those treated with BM-MSCs alone [107,108]. These findings can be explained by the anti-oxidant and anti-inflammatory effects of atorvastatin and the hematopoietic effects of DPO, respectively, both of which aid the therapeutic effects of BM-MSCs by creating a better organ microenvironment for their survival and function [107,108]. However, despite these studies investigating various approaches to enhance the survival, anti-inflammatory, and/or angiogenic effects of BM-MSCs, very few of them have addressed the anti-fibrotic effects of BM-MSC-based therapies, which are important in the context of CKD, as fibrosis progression has been shown to directly correlate with the degree of renal dysfunction [14]. More recently, evidence has emerged of a novel approach to improve the fibrotic microenvironment of chronically injured kidneys into which BM-MSCs can be administered to improve their survival, migration to site(s) of damage, and reparative efficacy. Recent studies have demonstrated that combining BM-MSCs with the anti-fibrotic and renoprotective agent serelaxin [20,71,72,78] might provide a more promising alternative (to the strategies described above) to enhance the therapeutic efficacy of BM-MSCs as a treatment for CKD, which is discussed further in the next section.

### Combining BM-MSCs with the Anti-Fibrotic Agent Serelaxin

Relaxin is an endogenous 6kDa peptide hormone that is mainly produced by the corpus luteum (ovary) during pregnancy and in lower quantities by the brain, heart and kidney to mediate adaptive hemodynamic changes during pregnancy (enhancing cardiac output, renal blood flow, and arterial compliance) [109,110,111]. Relaxin is a member of the relaxin family of peptide hormones, which consists of three relaxin peptides found in the human body, the products of human gene-1 (H1), gene-2 (H2), and gene-3 (H3), relaxin, respectively [109,112]. Of these three relaxin peptides, H2 relaxin is the major stored and circulating form of human relaxin in the body [113]. Most other species, including rodents, only contain two relaxin peptides, relaxin and relaxin-3, the species equivalents of H2 relaxin and H3 relaxin, respectively. This review focuses on H2 relaxin and its species equivalent relaxin peptide, which is referred to as RLX unless otherwise stated.

Endogenous relaxin deficiency in mice has been found to be associated with impaired development of several reproductive organs, leading to infertility in males [114], and an age-related progression of fibrosis in several non-reproductive organs including the lung, heart, kidneys, and skin [115]. This led to renal cortical thickening and impaired kidney function in male mice [116,117], which was attributed to the higher levels of testosterone found in males in contrast with the protective effects of estrogen in females [118]. Based on these findings, which identified endogenous relaxin as a naturally-occurring regulator of collagen turnover, a recombinant form of human relaxin-2 (also known as serelaxin; which is also referred to as RLX) was developed to evaluate the anti-fibrotic potential of RLX, which was found to be bioactive in several other species including rodents [119]. RLX has consistently demonstrated promising and rapidly acting anti-fibrotic efficacy in various experimental CKD models regardless of etiology, by either preventing fibrogenesis or effectively attenuating established tubulointerstitial fibrosis and glomerulosclerosis [71,72,78,120,121,122,123,124,125], leading to improved kidney function.

**Figure 5:**
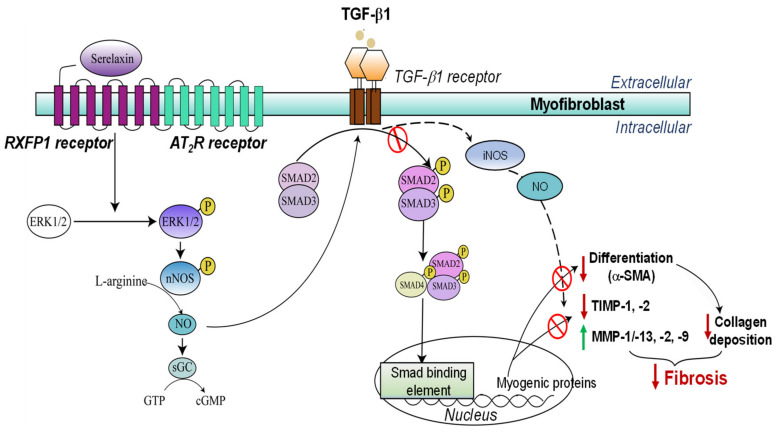
The signal transduction mechanisms underlying the anti-fibrotic effects of RLX in myofibroblasts. Upon binding to RXFP1 on myofibroblasts and remodelling through RXFP1 and RXFP1-AT_2_R crosstalk (when both receptors are adequately expressed), RLX inhibits the TGF-β1/Smad2 axis and the ability of Smad2 to interact with pro-fibrotic Smad3 and Smad4; this is required for the TGF-β1-induced promotion of myofibroblast differentiation and ECM production. As TGF-β1 promotes TIMP activity and inhibits MMP activity, the RLX-induced suppression of TGF-β1 ameliorates the TGF-β1-induced upregulation of TIMP-1 and -2 and promotes the expression and activity of various collagen-degrading MMPs (MMP-1/-13, -2, -9) from myofibroblasts. Figure adapted from [131].

The anti-fibrotic effects of RLX occur via remodelling through its cognate G protein-coupled receptor, relaxin family peptide receptor-1 (RXFP1), or through the crosstalk that can occur between RXFP1 and the Ang II type 2 receptor (AT_2_R) [126,127], particularly through a RXFP1-phosphorylated extracellular signal-regulated kinase (ERK)1/2 (pERK1/2)–neuronal nitric oxide (NO) synthase (nNOS)–NO-soluble guanylate cyclase (sGC)–cGMP-dependent pathway to suppress Smad2 phosphorylation (pSmad2) [128,129,130,131,132]. This negates the ability of Smad2 to interact with Smad3 or with Smad4, which promotes the pro-fibrotic effects of TGF-β1 on myofibroblast differentiation [133] and myofibroblast-mediated aberrant ECM deposition (Figure 5). Through the same pathway and the additional stimulation of inducible NOS (iNOS)–NO activity, RLX also promotes the expression and activity of various collagen-degrading MMPs to facilitate ECM degradation [129,131] (Figure 5). In addition to its anti-fibrotic actions, RLX exerts angiogenic effects via its stimulation of VEGF and bFGF expression and activity and induces vasodilation by activating the endothelin receptor–NO signalling pathway [111,134,135]. Furthermore, RLX can therapeutically reduce the influx of various immune cells including granulocytes such as mast cells and neutrophils [136] in preclinical models of ischemia/reperfusion injury and inhibit macrophage infiltration in mice subjected to obstructive nephropathy- and hypertension-induced renal fibrosis [71,72]. The anti-inflammatory actions of RLX are additionally modulated by suppressing immune-cell-derived pro-inflammatory cytokines (such as IL-1β, IL-6 and TNF-α) and the expression of TNF-α-stimulated MCP-1 and vascular cell adhesion molecules (VCAM-1) in the kidneys [124,137]. These pleiotropic organ-protective effects contribute significantly to the anti-remodelling and tissue-reparative effects of RLX in chronic disease settings [20].

The combined effects of RLX (0.5 mg/kg/day; a dose frequently used to induce its anti-fibrotic and other organ-protective effects [71,72,78,126,128]; subcutaneously (sc) administered via slow-release osmotic minipumps) and BM-MSCs (1 × 10^6^/mouse, delivered via the intrarenal vein 15–30 min after RLX administration) completely attenuated fibrosis progression in normotensive mice subjected to one week of unilateral ureteric obstruction (UUO)-induced tubulointerstitial fibrotic disease in vivo [71] (Table 4). This was to a greater extent than the effects of BM-MSCs (which did not induce any anti-fibrotic effects alone) or RLX (which only partially attenuated kidney fibrosis) alone over the 7-day treatment period. The same study also demonstrated that RLX directly enhanced the proliferation (at 1 ng/mL) and migration (at 10 or 100 ng/mL) of BM-MSCs in vitro by binding directly to RXFP1 receptors expressed on the surfaces of BM-MSCs. It was therefore proposed that RLX might enhance BM-MSC migration and survival post-transplantation in vivo, thereby more effectively attenuating fibrosis progression than the effects of BM-MSCs alone.

In separate studies conducted in hypertensive mice subjected to uninephrectomy followed by implantation of a slow-release deoxycorticosterone acetate pellet and saline to drink (1K/DOCA/salt) for three weeks [72], the combined effects of RLX (0.5 mg/kg/day; sc administered via slow-release osmotic minipumps) and BM-MSCs (1 × 10^6^/mouse; iv administered via a tail-vein injection 15–30 min after RLX administration) significantly attenuated established interstitial kidney fibrosis and glomerulosclerosis to a greater extent than either treatment alone when administered on day 14 post-injury (Table 4). More excitingly, BM-MSCs combined with RLX provide broader renoprotection and anti-fibrotic efficacy than the clinically used ACEi, perindopril [72], or RLX- and/or BM-MSC-derived exosomes (25 μg/mouse, equivalent to the therapeutic impact of 1−2 × 10^6^ BM-MSCs/mouse; iv administered via a tail-vein injection [78]), despite the increased interest in and manufacturing advantages of using stem-cell-derived exosomes as therapeutics. Importantly, this novel combination therapy was able to attenuate SBP to a similar extent to that achieved by perindopril [72] or the aldosterone receptor blocker spironolactone [78], owing to the anti-hypertensive effects of the BM-MSCs (discussed above). Furthermore, only the combined effects of RLX and BM-MSCs, but not RLX and/or BM-MSC exosomes, were able to significantly reduce the 1K/DOCA/salt-induced increase in kidney injury molecule (KIM)-1 associated tubular epithelial damage and restore the 1K/DOCA/salt-induced loss of peritubular capillary density, highlighting the broader renoprotective effects offered by the combined effects of RLX and BM-MSCs.

To enhance the translational impact of this combined therapy, its ability to stimulate endothelial progenitor cells (EPCs) isolated from the blood of stage V ESKD patients was evaluated in vitro (Table 4). EPCs play key roles in the regeneration of the endothelial lining of blood vessels but are progressively lost during CKD, which correlates with adverse organ outcomes and related mortality [138]. Excitingly, it was found that the combined effects of BM-MSC-derived conditioned media (CM) and RLX (10 ng/mL) significantly promoted EPC viability, capillary tube formation, and wound closure in vitro after 24 h; to a greater extent than BM-MSC-CM (wound closure) or either therapy (capillary tube formation) alone [139]. Collectively, these findings highlighted the enormous therapeutic value of utilising the combined effects of RLX and BM-MSCs as a treatment for patients with normotensive (via the antifibrotic and tissue-reparative effects of the combined treatment) or hypertensive (via the anti-hypertensive, antifibrotic, and tissue-reparative effects of the combined treatment) CKD.

**Table 4:** Key findings from preclinical studies conducted to date that have evaluated the combined therapeutic effects of RLX and BM-MSCs in experimental models of CKD.

Models	Treatment Regime	Main Outcomes	Reference(s)
**In vitro**			
BM-MSCs	Treated with 1–100 ng/mL RLX for 24 h or 72 h	↑ BM-MSC proliferation at 1 ng/mL after 72 h ↑ BM-MSC migration at 10 and 100 ng/mL after 24 h	[71]
Human EPCs isolated from the blood of stage V ESKD patients	Combination of 25% BM-MSC-derived conditioned medium (CM) + 10 ng/mL RLX	↑ EPC proliferation and wound closure over 24 h ↑ EPC capillary tube formation over 4 h	[139]
**In vivo**			
UUO-induced obstructive nephropathy (7 days)	RLX (0.5 mg/kg/day) via sc implanted osmotic minipumps + iv injection of BM-MSCs (1 × 10^6^ per mouse); immediately after UUO	↓ Tubular epithelial injury ↓ Macrophage infiltration ↓ Myofibroblast accumulation ↓ Collagen concentration ↑ MMP-2 activity	[71]
1K/DOCA/Salt-induced hypertension (21 days)	RLX (0.5 mg/kg/day) via sc implanted osmotic minipumps + iv injection of BM-MSCs (1 × 10^6^ per mouse); on day 14	↓ SBP ↓ Tubular epithelial injury ↓ Inflammation, fibrosis, and proteinuria ↑ Creatinine clearance ↑ Peritubular capillary density * Provided broader reno-protection than RLX and/ or BM-MSC-derived EXO, perindopril, or spironolactone	[72,78]

## 5. Concluding Remarks

CKD has placed a huge socioeconomic burden on numerous healthcare systems worldwide, with kidney fibrosis being the major hallmark of disease progression. However, due to the multifactorial nature of fibrosis and the fact that current treatments only target the specific component of fibrosis and offer symptomatic management disease pathology, a novel therapy for CKD patients is urgently required. As fibrosis also acts as a barrier to the viability, migration, and integration of implanted stem cells, combining the anti-fibrotic and organ-protective effects of RLX with RXFP1-expressing BM-MSCs possesses several properties of an effective anti-fibrotic and renoprotective cell-based therapy. Despite the exciting potential of this combined therapy, as discussed above [71,72,78,139], there are some issues with its potential clinical application, which should be addressed in future investigations. Firstly, the short (7-day) treatment period investigated in the above-mentioned studies was intentionally chosen, as BM-MSCs have been found to be cleared from the damaged kidneys within 7 days of treatment, despite exerting longer-term effects well after they have been cleared [140,141]. However, longer-term studies (following repeated administration of BM-MSCs) are required to address the question of whether the anti-hypertensive, anti-inflammatory, and anti-fibrotic effects of this combination therapy can be maintained over a longer-term period. Secondly, the optimal dose and infusion method of RLX and BM-MSCs as a combined therapy should be established to achieve their successful transition in clinical settings. Unlike in animal models (which require sc administration of RLX followed by iv delivery of BM-MSCs), a non-invasive infusion approach should be applied to administering this combined therapy to humans. One potential means of addressing this latter point could be to genetically modifying BM-MSCs to over-express RLX, such that the BM-MSCs act as vehicles that can deliver the therapeutic impact of the combined effects of BM-MSCs and RLX, which would allow for the combined effects of both therapies to be administered simultaneously via a single mode of administration. Furthermore, the longer-term safety and efficacy of combining BM-MSCs and RLX should be evaluated in clinical studies involving CKD patients in the future. Addressing these challenges will hopefully lead to the clinical application of BM-MSCs and RLX as a stand-alone or adjunct therapy for patients suffering from CKD.
